# Leaders like us: landscape of physical activity leadership programmes for underserved young women in England

**DOI:** 10.1186/s12966-026-01886-0

**Published:** 2026-03-14

**Authors:** Jamie Crowther, Sufyan Abid Dogra, Sally Barber, Nazaket Ali, Jennifer Hall

**Affiliations:** 1 Born in Bradford, Bradford Teaching Hospitals NHS Foundation Trust, Bradford, United Kingdom; 2 Bradford Childrens and families trust, Bradford, United Kingdom; 3 Bradford Centre for Qualitative Research, Bradford, United Kingdom

## Abstract

**Background:**

Despite growing advocacy for youth-led and inclusive physical activity provision, there remains limited evidence on how to effectively support underserved young women aged (16–25) and those from minoritised genders into Physical Activity leadership roles.

**Methods:**

We used a two-phase sequential design. Phase 1 involved systematic mapping of 53 PA leadership programmes in England to describe programme focus, target populations, delivery models and stated outcomes. Phase 2 comprised seven online focus groups with 41 practitioners involved in designing, delivering or overseeing such programmes. Mapping data were summarised using descriptive statistics and narrative synthesis; focus group data were analysed using hybrid inductive–deductive framework analysis informed by Critical Positive Youth Development and feminist–intersectional lenses.

**Results:**

Only a minority of mapped programmes were explicitly designed for girls and young women from underserved groups. Programmes most commonly operated through school- or club-based delivery, using modular learning, mentoring and cascaded models, with fewer incorporating financial support, digital delivery or clearly defined progression routes. Practitioners described how recruitment frequently relied on institutional gatekeepers and digital communication, raising concerns about who is reached and who is missed. They emphasised the importance of relational and culturally grounded recruitment and support, care-oriented infrastructures, and broader conceptualisations of leadership that include advocacy, representation and community influence.

**Conclusion:**

Combining systematic mapping with practitioner insights provides an integrated descriptive and interpretive picture of current provision. The findings indicate uneven targeting of underserved girls and young women and variable support for their progression into leadership roles. Enhancing equity in PA leadership development will require attention to recruitment practices, relational support and progression pathways that recognise diverse forms of youth leadership.

## Introduction

Inequalities in young people’s Physical Activity (PA) participation persist globally, with girls, young women, and those from lower-income and racially minoritised communities consistently least active [1, 2]. These disparities matter not only for physical and mental health [3, 4], but because participation builds confidence, social capital, and leadership skills. Limited PA access reinforces structural inequalities and restricts pathways to community engagement, education, and employment [5, 6].

Building on previous research [7], we posit that diversification of the PA workforce by upskilling underserved young women and gender diverse youth as PA leaders can strengthen the PA landscape. Such programmes can improve access and support population-level PA by increasing representation and supporting the development of a youth PA workforce that is culturally, politically and socially responsive [8, 9]. This harnesses relatability to help diverse groups of young people to feel connected to PA [7, 10–12].

National and international strategies, including Sport England’s *Uniting the Movement* [13] and the International Society for PA and Health’s [14] call for workforce diversification, position inclusive leadership development as a key to addressing inactivity disparities. A diverse PA workforce is increasingly recognised as a social justice issue, where relatability and representation are foundational mechanisms of engagement [15, 16]. Leadership in PA concerns not only individual skills but who is visible, who holds influence, and whose experiences shape decision-making.

We draw on Critical Positive Youth Development (CPYD) and feminist–intersectional leadership perspectives to frame youth PA leadership as relational, context-dependent and potentially transformative [17, 18]. CPYD emphasises quality and equity of developmental settings, and how resources, power and opportunities are distributed, rather than assuming all leadership experiences benefit youth. Feminist and intersectional lenses highlight how gendered, racialised, classed and other intersecting structures shape who is recognised as ‘leaders’, what leadership is valued, and whose labour remains invisible. Together, these lenses guide our focus on programmes design, reach, support mechanisms and the progression they offer, and how they may reproduce or disrupt existing inequalities.

Despite increasing advocacy, youth PA leaders’ development is under-researched, with little known about program mechanisms and social justice alignment [19]. There is particular lack of review regarding inclusion of young females from underserved groups. Existing work examines youth leadership generally, or focuses on participation rather than leadership, and rarely combining systems-level provision overview with in-depth insight into how inclusion, support and progression are understood in practice. As a result, we lack integrated understandings of PA leadership opportunities for underserved girls and young women, how programmes identify and engage them, and how far they reflect CPYD and intersectional social justice principles. Phase One of the Leaders Like Us project sought to generate an integrated picture of current provision and practice, combining systematic mapping with practitioner focus groups. We aimed to:

1. Descriptively map PA leadership programmes in England that engage underserved girls and young women, including how they identify, recruit and support participants and what outcomes they intend; and.
2. Collate practitioner-generated recommendations for strengthening equity in the design, delivery and progression routes of young PA leader programmes.

This study makes three main contributions. Methodologically, it integrates systematic mapping with practitioner focus groups, generating an integrated picture of provision and practice. Conceptually, it develops an equity-focused understanding of youth PA leadership for underserved girls and young women, foregrounding CPYD and feminist–intersectional lenses to move beyond narrow, coaching-based models. Practically, it identifies principles for recruitment, support and progression of underserved young women to inform policy and programme design.

## Methods

### Study context

This paper forms part of Leaders Like Us: a multi-phase research programme co-producing a framework to help young PA leaders’ programmes to better identify, recruit and support young women aged 16–25 from underserved groups. The wider Leaders Like Us programme adopts a co-productive approach, combining systematic mapping, qualitative inquiry through focus groups, and co-design to generate a practical national framework for inclusive PA leadership development. This paper reports on Phase One, providing the empirical foundation for later stages of the programme.

### Clarification of key terms

#### Leadership

We use *leadership* broadly to encompass formal and informal roles where young people take initiative, influence others, contribute to collective goals, or act as agents of change within PA settings. This includes coaching, facilitation, mentoring, organising, advocating, and role-modelling [20, 21]. We move beyond narrow, hierarchical, or credential-based definitions, instead recognising leadership as relational, contextual, and potentially transformative [22]. We acknowledge traditional notions can marginalise young people whose leadership do not align with dominant expectations, and our use of the term holds space for diverse expressions of youth agency, voice, and influence [23, 24].

#### Underserved groups

Understandings of ‘underserved groups’ are varied and often ill-defined, frequently overlapping with terms such as marginalised, underrepresented, and seldom-heard [25, 26]. We adopt the term intentionally to foreground systemic failure. Underserved groups are systematically denied access and opportunity due to structural barriers and chronic underinvestment [27]. While acknowledging limitations and potential imprecision of the term, it reflects our commitment to placing accountability on systems and institutions rather than on individuals or communities and aligns with our understanding that exclusion is neither neutral nor accidental, but actively produced through structural power imbalances, institutionalised discrimination, and embedded bias.

### Study design and theoretical framing

This study employed a two-phase design with two aims: Phase 1 comprised Systematic mapping of existing young PA leadership programmes to address aim 1 by providing a descriptive overview of what programmes exist in England, who they target, and how they are structured. Phase 2 comprised practitioner focus groups to extend aim 1 and address aim 2 by generating interpretive, practitioner-informed insights and recommendations about how identification, recruitment, support and progression can be made more equitable in practice. Both phases were interpreted through CPYD and feminist–intersectional lenses, which foreground questions of power, representation and distribution of resources and opportunities.

Ethical approval was granted by the Research Ethics Panel, University of Bradford, UK (ethics application: E1274, 26/11/2025).

### Critical positive youth development and intersectionality as analytic lenses

We framed our investigation through Critical Positive Youth Development (PYD): acknowledging that all youth have the capacity to thrive when provided with the right contextual supports [28], and that leadership is more than individual capacity [29, 30] but influenced by structural inequality, and shaped by power, exclusion, and systemic constraint [17, 31, 32]. Leadership development must address injustice, be cultural affirming and politicised, not simply focused on individual skill-building.

Alongside CPYD, intersectionality [33] served as a guiding analytic lens. We examined how gender, ethnicity, class, faith, disability, and other intersecting social positions shaped access, support, and recognition within leadership programmes. Rather than treating identity as additive categories, we explored how overlapping power systems shaped which young females were seen, supported, and developed as leaders. Together, CPYD and intersectionality informed both the analytic approach and design principles of this study, and Leaders Like Us more broadly.

#### Systematic mapping of young leaders’ programmes

Systematic mapping was employed to understand the landscape of young PA leadership programmes for young women aged 16–25 in England. Between October 2024 and April 2025, using published methods [34], we systematically mapped of programmes designed to develop PA leadership among underserved girls and gender-diverse youth in England. We sought to maximise completeness by combining formal searches with snowballing through sector networks and national organisations, continuing searches until repeated checks yielded no new programmes. Nonetheless, we recognise some small-scale or less visible initiatives may not have been identified. Table 1 below outlines the inclusion criteria and rationale.

**Table 1:** Systematic mapping inclusion criteria

Inclusion criteria	Rationale
Been intentionally designed to develop some aspect of PA leadership, as defined by the delivering organisation.	Leadership is often a secondary or unintended outcome of youth participation in sport and PA. This study focused on programmes explicitly aiming to develop PA leadership, where leadership was a central, stated goal rather than by-product, allowing deeper insight into how leadership was defined, operationalised, and supported by delivering organisations.
Included youth aged 16–25;	The project focused on young women aged 16–25, a critical transitional period as they begin considering post-school pathways.
Been situated (a)Withernsea, Birmingham and Solihull, Bradford, Greater Manchester, Doncaster, Redcar & Cleavland and Middlesborough, Essex, Hackney, Southall, Greater Exeter, Pennine Lancashire, and Calderdale.) within one of the original Sport England Local Delivery Pilot locations or (b) were delivered by a national organisation (e.g., a national governing body).	Focusing on delivery within Sport England’s Local Delivery Pilot areas aligned the mapping with efforts explicitly designed to tackle structural barriers to PA participation through deep community engagement. Including programmes delivered by national organisations enabled us to capture initiatives embedded within broader strategic agendas and national frameworks for youth and community sport
Been delivered 2014–2025.	Limiting the timeframe to programmes delivered since 2014 was pragmatic to manage scope, yet ensured the mapping captured recent shifts in sport and PA policy, particularly the turn toward social justice and inclusion. This period saw a move from short-term interventions toward more participatory and equity-oriented approaches, reflected in strategic developments such as Sport England’s Towards an Active Nation (2016) and Uniting the Movement (2021) and the intersectional reframing of This Girl Can (2018).

^a^Withernsea, Birmingham and Solihull, Bradford, Greater Manchester, Doncaster, Redcar & Cleavland and Middlesborough, Essex, Hackney, Southall, Greater Exeter, Pennine Lancashire, and Calderdale

A three-pronged search strategy identified relevant programmes from: (a) grey literature (e.g., organisation websites, public databases, social media), (b) grey information (e.g., direct outreach to relevant organisations and networks, implementing a snowball method [35] (where appropriate), and (c) published literature. For the published literature, structured searches were conducted using online database (e.g., Google Scholar) with keywords such as ‘girls sport leadership’, ‘youth physical activity leadership’ etc. Published literature provided contextual background but was largely evaluative and did not offer additional descriptive insight into programme context, mechanisms, or composition beyond grey sources.

Grey literature was accessed through targeted online searches of organisation websites and social media. Organisations and programmes were identified via existing networks, online visibility and the research team’s prior knowledge, supported by Google and social media searches (LinkedIn, X, Facebook). Relevant practitioners were contacted by email to arrange a brief screening call, which confirmed programme eligibility and clarified contribution to mapping. Practitioners could provide information verbally during an online meeting or complete a bespoke mapping form, a flexible approach designed to maximise accessibility and accommodate different capacities for engagement.

Using Excel, a standardised data extraction form was developed and piloted on a subsample (*n* = 5) to assess usability and relevance. Based on piloting, minor adaptations clarified wording and improved consistency in data entry. The revised extraction tool was rolled out across all eligible programmes. Extracted data included:

- Delivery period.
- Programme aims.
- Participant inclusion criteria.
- Whether the program focused on females and gender-minority youth, and if so, why.
- Whether the program focused on specific underserved groups, and if so why.
- Programme delivery location.
- Program’s delivery approach.
- How youth were identified and recruited.
- How youth are supported to maintain engagement.
- Programme learning outcomes.
- Recommendations for developing young females as PA leaders.

### Focus groups

Focus groups facilitated understanding of how and why programmes were designed and delivered the way they were and built on learnings from systematic mapping. The focus groups aimed to:

- Explore identification/recruitment approaches, support mechanisms and learning outcomes in previous programmes.
- Reflect on systematic mapping findings.
- Elicit practitioner perspectives on effective approaches, challenges and needs for programmes serving underserved females.

We used purposive sampling to recruit practitioners involved in the design, delivery or strategic oversight of programmes. We sought diversity in roles (e.g. community/grassroots delivery staff, workforce and skills lead, senior leaders, and faith/community-based practitioners) and contexts (e.g. local authorities, charities, national governing bodies, educational settings). Participants were identified through mapping, existing networks and partner recommendations, and invited via email to reflect on mapped programmes and discuss equity-focused leadership development.

Focus groups were conducted via Microsoft Teams, recorded with informed consent and transcribed using Microsoft’s transcribe function. Transcripts were cleaned and anonymised by the research team.

### Analysis strategy

#### Systematic mapping analysis

Programmes were charted in a standardised Excel data extraction tool. Structured variables (e.g. delivery period, setting, target groups) were summarised using descriptive statistics. Qualitative fields (e.g. free-text descriptions of recruitment, support, outcomes and recommendations) were organised into deductive categories derived from the study aims (recruitment, support, outcomes, recommendations). Within each category, inductive coding identified recurring mechanisms which were grouped into thematic categories. Coding decisions were refined through team discussion to preserve important equity-related nuances. These analyses addressed Aim 1 by describing the distribution of programme characteristics and mechanisms; we did not evaluate programme effectiveness of quality.

#### Qualitative data analysis

Hybrid inductive-deductive framework analysis was used for focus group and mapping data [36]. Transcripts were mapped onto deductive domains (recruitment, support, outcomes, recommendations), reflecting our research aims. Within each domain, inductive subthemes capture patterned meanings in practitioners’ accounts and in programme descriptions. We compared mapped programme categories and focus group subthemes, examining convergence, diverged and complementary insight. This interpretive integration, which addressed Aim 2, is presented in the thematic qualitative findings and in the discussion.

### Positionality statement

The research team JC and JH are academics specialising in youth PA, gender equity and community-based programme development. This afforded familiarity with policy and delivery structures but also positioned us as relatively privileged compared to many of the underserved girls and young women discussed in the data. Our prior work on CPYD and PA inclusion informed the decision to focus on equity, representation and power, potentially sensitising us to structural constraint and practitioner labour. We made reflexive notes after focus groups, kept analytic memos during analysis and held regular bi-weekly discussions comparing interpretations, seeking disconfirming examples examples and considering how our assumptions and roles might shape analysis. 

#### Narrative synthesis of qualitative systematic mapping data

Narrative synthesis [37] was applied to qualitative descriptions for systematic mapping to identify trends, gaps, and delivery models reflecting Leaders Like Us focus on social justice, youth leadership, and inclusion. Mapping and focus group were collected and analysed separately; mapping provided structural overview while focus groups added depth and nuance. Findings are presented across the four domains: (1) Identification and recruitment, (2) Support, (3) outcomes, and (4) recommendations.

## Results

In keeping with our two-phase design, we first present descriptive findings from the systematic mapping (Aim 1), which outline what PA leadership programmes currently exist, who they target and how they are structured. We then present qualitative findings integrating programme descriptions with practitioner accounts from focus groups (Aims 1–2), which illuminate how identification, recruitment, support and progression are understood and experienced in practice.

### Quantitative results – descriptive overview of mapped programmes (Aim 1)

#### Programme characteristics

We identified 53 young leaders’ programmes that met the inclusion criteria. Eleven programmes had a female-only design. Five of these were delivered by a sport-specific National Governing Body to increase female representation in a particular activity; five aimed to increase female representation in PA leadership more broadly; and one did not specify a rationale for its single-sex design. Outside these 11 programmes, one initiative aimed for at least 50% female representation, and one programme originally designed as female-only later widened its eligibility to include males.

#### Programme focus and target population

**Fig. 1:**
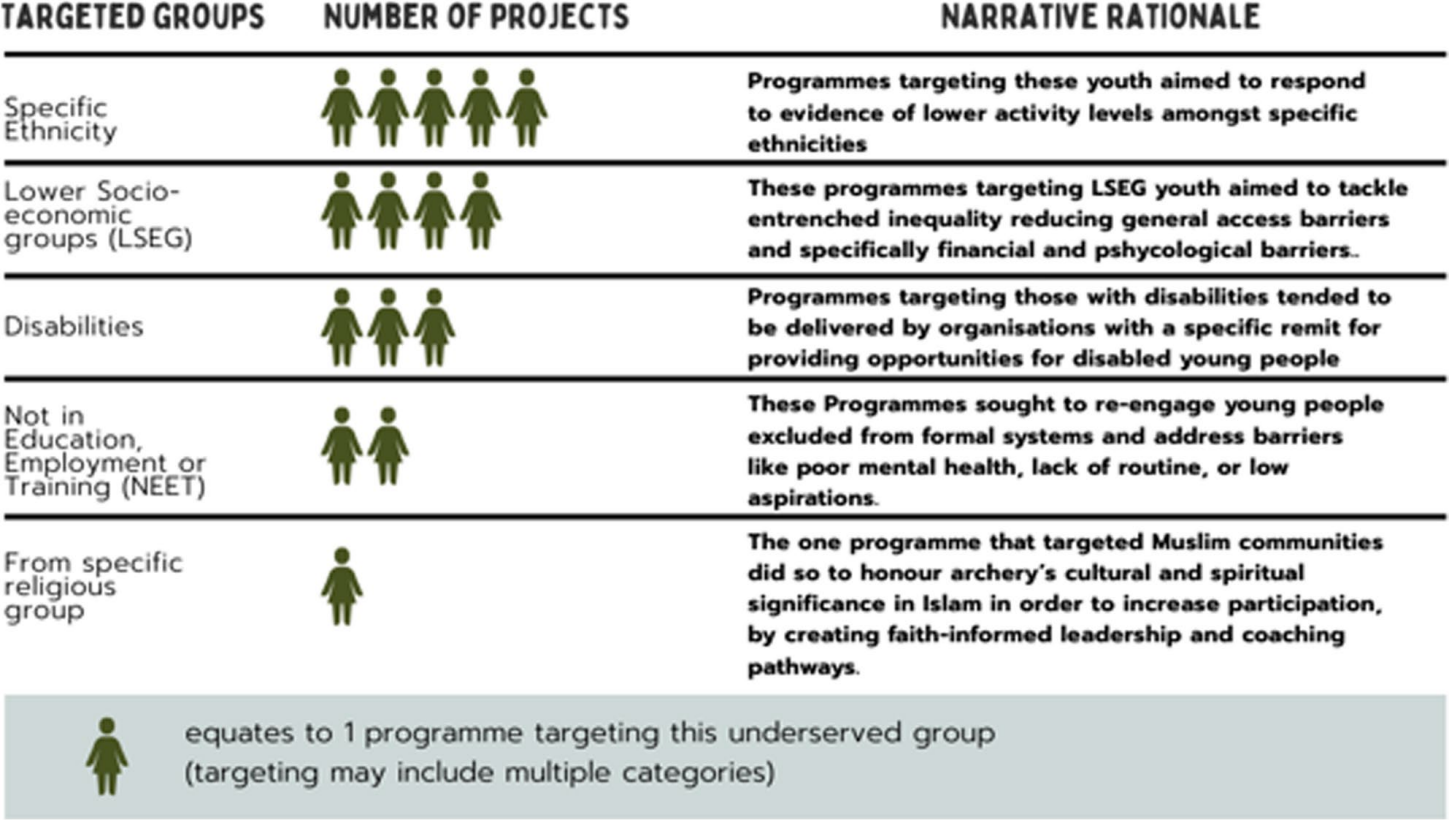
Target Underserved Groups across Youth Leadership Programmes

Mapped programmes varied in whether they explicitly targeted underserved groups or were framed more generally. Figure 1 summarises the target populations reported across the 53 programmes. Several programmes focused on inequalities related to ethnicity, socio-economic background or disability, while a smaller number explicitly targeted young people who were not in education, employment or training (NEET) or particular religious groups. Programmes often name more than one target group, reflecting overlapping foci, and meaning individual programmes could be madded in multiple categories.

#### Delivery setting and mechanisms

**Fig. 2:**
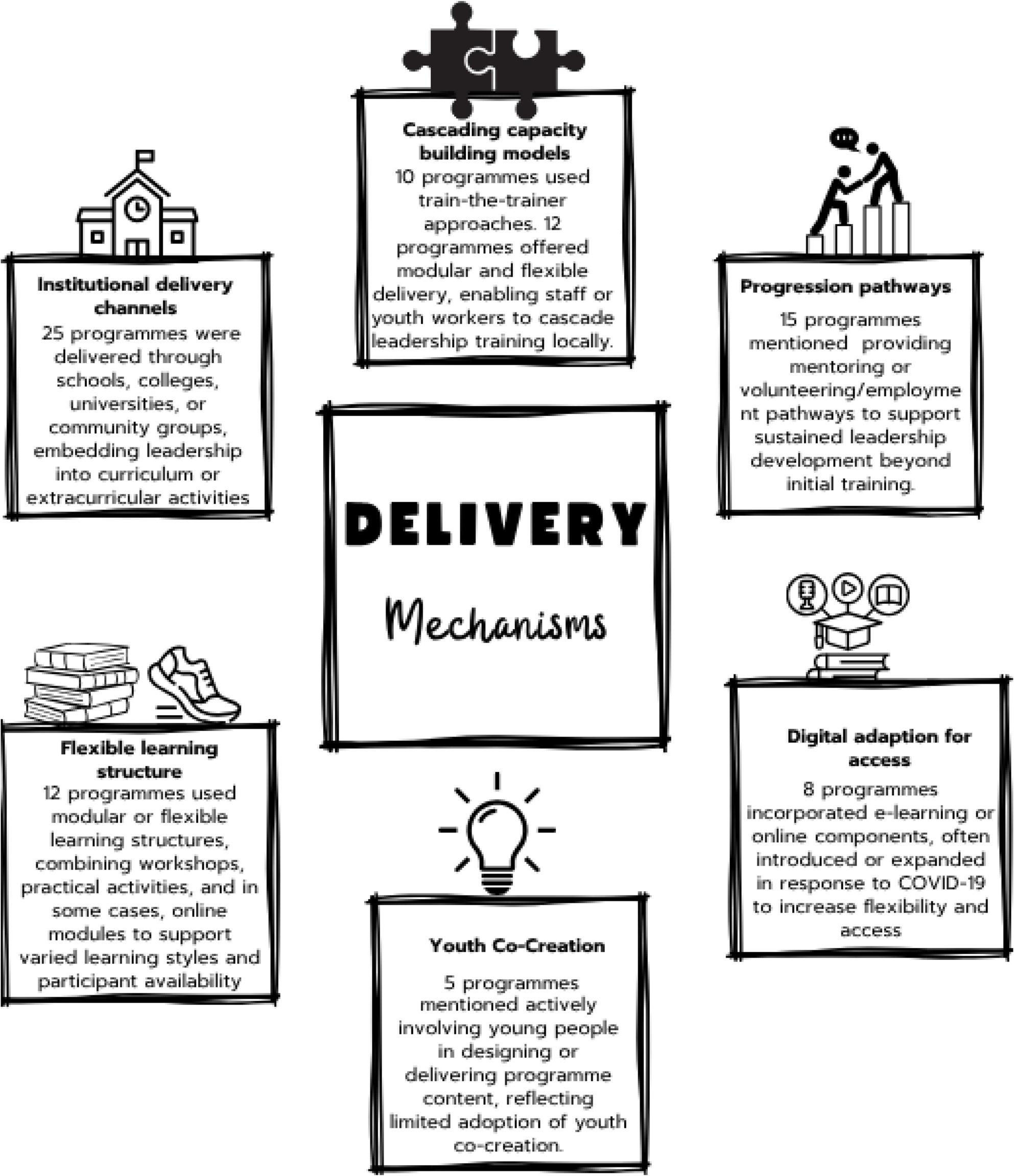
Delivery Mechanisms Used in Youth Leadership Programmes

Programmes were delivered across a range of settings. Many were embedded within existing infrastructures such as schools, colleges and community organisations. Others were delivered through club- or community-based provision, or by national organisations operating across multiple localities. Figure 2 summarises the reported delivery mechanisms. Common components included modular learning, mentoring and peer-support structures, and cascaded models in which trained young leaders subsequently supported local delivery. Some programmes incorporated digital elements (for example, online training or blended learning formats), particularly in the post-COVID period.

#### Temporal distribution of programme initiation

Programmes began at different points between 2014 and 2025 (Table 2). Seven programmes started before 2014, three between 2014 and 2016, eight between 2017 and 2019, eleven between 2020 and 2022, and three between 2023 and 2025, with start dates unknown for seven programmes. The largest number of programmes (*n* = 11) began between 2020 and 2022, with smaller numbers initiated in earlier and later periods.

**Table 2:** Table of youth leadership programme delivery trends

Programme start period	Number of programmes beginning in that period
Pre-2014	7
2014–2016	3
2017–2019	8
2020–2022	11
2023–2025	3
Unknown/ No Data	7

Focus Group participants characteristics

Table 3 summarises participant characteristics and focus group composition.

**Table 3:** Focus group characteristics table

Characteristic	Detail
Number of focus groups	7
Duration of focus groups	90 min
Total participants	41
Average age	38.7 ± 8.9 years
Self-reported ethnicity	35 white British 2 British Pakistani 2 Black British 1 British Asian 1 Indian
No. who self-reported Neurodivergence	5
No. who self-reported disability	2
Median years of experience working in youth PA leadership development with underserved groups	15 years (range 2–25)
Gender identity	63.4% Female (*n* = 26), 36.6% Male (*n* = 15)

Table 4 below outlines participant roles, classified to protect anonymity.

**Table 4:** Professional roles of focus group participants, grouped by leadership level and context of practice

Clustered focus group roles	Number
Senior leadership (e.g., directors, CEO’s)	11
Development/Programme officers (e.g., Inclusion roles, sport-specific development workers)	11
Workforce/Skills leads (e.g., Workforce development leads, policy leads)	12
Community/grassroots practitioners (e.g., Dance teachers, sports coaches)	6
Faith/Community leaders (Faith/community leaders, Academy manager)	2

### Practitioner perspectives on recruitment, support, outcomes and recommendations (Aims 2)

The following section presents qualitative findings organised into four themes: (1) recruitment and identification mechanisms; (2) support mechanisms; (3) programme outcomes; and (4) practitioner-informed recommendations for future practice.

### Theme 1: recruitment & identification mechanisms

**Fig. 3:**
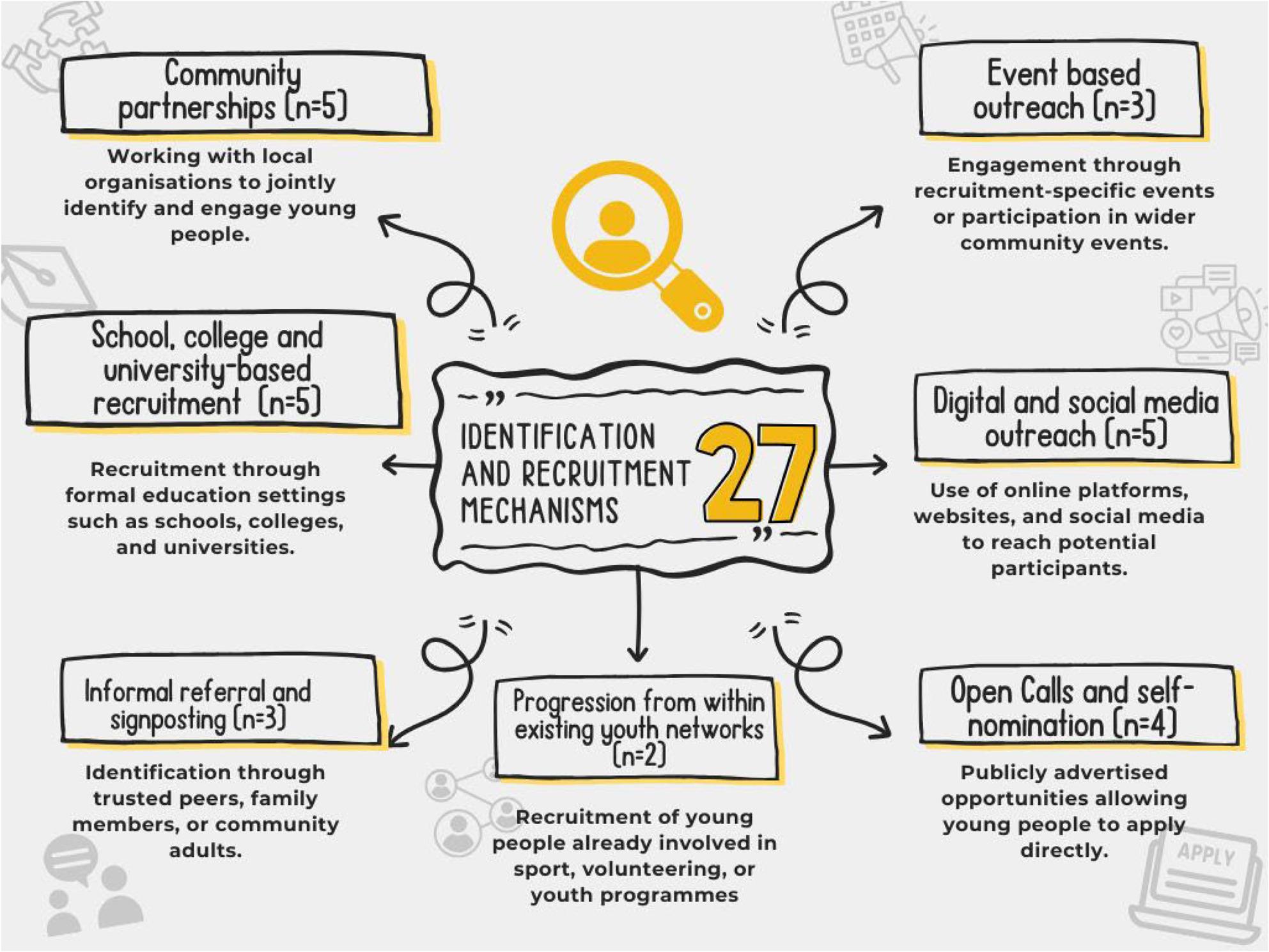
Identification and recruitment methods found during systematic mapping

Systematic mapping identified 27 recruitment and identification mechanisms clustered into seven categories (Fig. 3). These suggest reliance on institutional and digital networks, with limited emphasis on intentional progression. This context informed focus group discussions, where practitioners reflected on who is reached and who is overlooked.

#### Gatekeeping and the reproduction of leadership inequality

Practitioners described a reliance on recruiting resourced, visible youth connected to institutions, particularly schools and colleges. particularly schools, colleges and partner organisations. Though framed as pragmatic, this approach was critiqued for reinforcing exclusivity and reproducing inequalities:

*“You do end up with a pretty homogeneous kind of group… the same people doing the same volunteering…” [Senior leadership].*

Subjective selection by adult gatekeepers (teachers, staff, or youth workers) based on perceived ‘potential’ was seen to favour confident, well-known, or high-achieving individuals and marginalise quieter or less visible girls.

*That organisation just sees something in them… they think*,* that person’s got the potential.* [Workforce/skills lead]

*We rely on colleges… [young people are often recruited via a] tap on the shoulder from a member of staff… it goes back to the reason they are getting identified? It’s because they’re showing certain qualities or because they’re known really well.* [Development/programme officer].

These mechanisms embed classed, racialised and gendered assumptions about who looks like a “leader”, privileging extrovert, performance-orientated models of leadership over relational or community-embedded forms.

#### Barriers to open recruitment: digital exclusion and access inequality

Some programmes attempted open recruitment through posters, online platforms, and organisational promotion. However, digital and linguistic exclusion limit those who could respond:

“Some… didn’t have access to a mobile phone or a computer or access to a website… a huge barrier around engagement.” [Senior leadership.

Formal application processes (CVs, cover letters, and structured interviews) were also viewed as replicating institutional barriers common in education and misaligned with many underserved young women’s circumstances. In response, some programmes shifted towards low-barrier, relational-approaches:

“We don’t ask for application forms, CVs, or covering letters… just ask people to express an interest and provide a phone number… then have a softer conversation… You know we’re not looking for loads of coaching experience .” [Senior leadership].

This shift toward relational, low-barrier recruitment were framed as important for equitable access and youth agency.

#### Relational and culturally responsive recruitment strategies

Practitioners described emerging shifts toward relational, culturally competent recruitment, particularly when recruiting across ethnicity groups. Within this, trust-building with families, community leaders and faith organisations was seen as central:

“We engaged with that mosque… built up trust… that led to parents saying, could you do something for the girls?” [Senior leadership].

Recruitment was therefore framed as an ongoing process of cultural alignment and negotiation. Practitioners emphasised tailoring programmes to target groups’ interests and aspirations, challenging restrictive definitions of leadership:

“[There are] cultural nuances… we tailored the programmes to fit the girls… it’s about creating those opportunities that fit the needs of the people.” [Senior leadership].

Taken together, Theme 1 illustrates how recruitment practices can reinforce or challenge inequalities, depending on whether they move from gatekeeping towards relational, culturally grounded and youth-centred approaches.

### Theme 2: engagement and support mechanisms

**Fig. 4:**
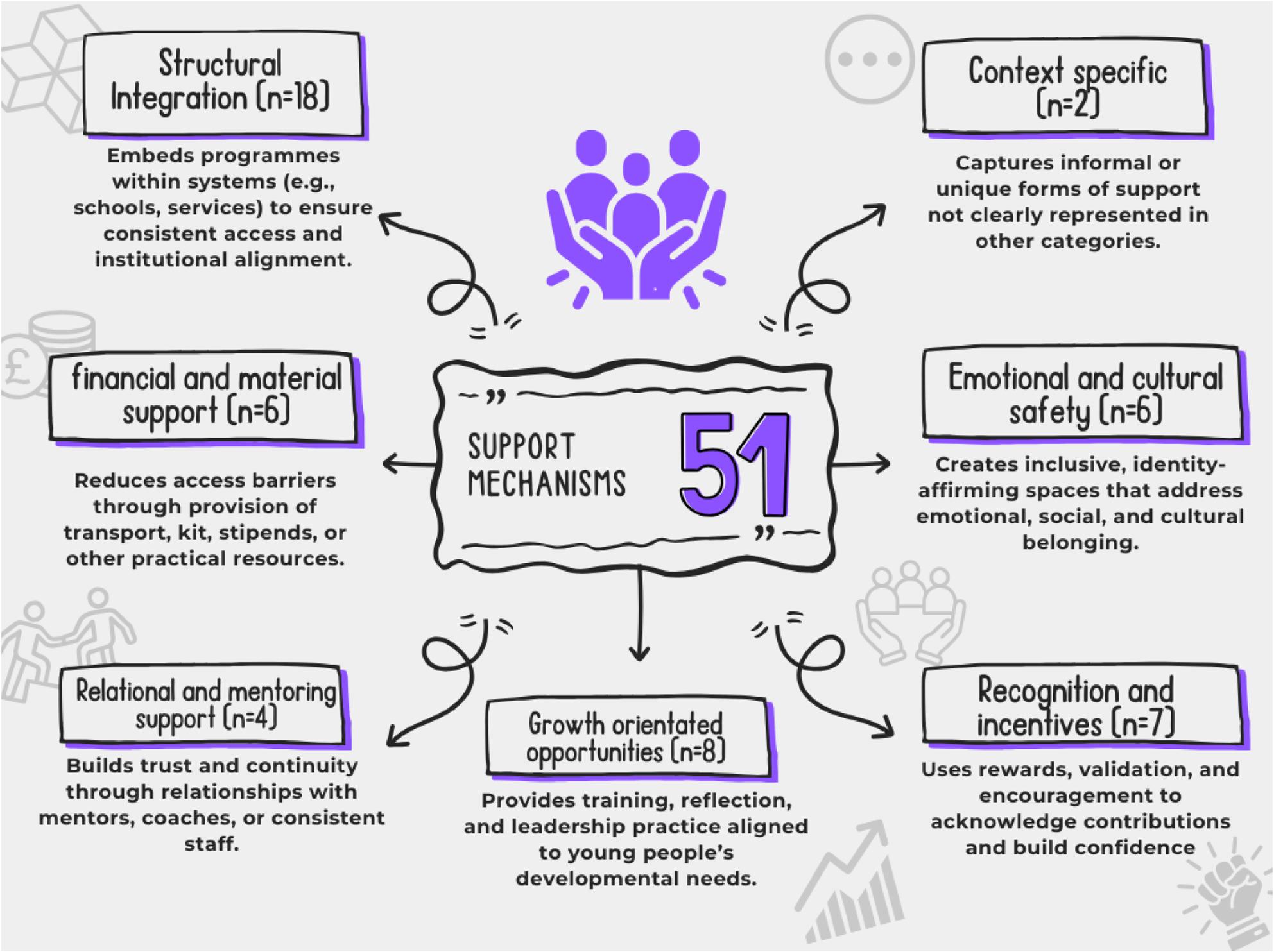
Support mechanisms found during systematic mapping

Systematic mapping identified 51 support mechanisms across seven themes. Most prevalent was structural integration (embedded leadership into existing institutions), alongside growth orientated opportunities and recognition/incentives. Financial support, emotional and cultural safety, and relational mentoring appeared less consistently. Overall, structural integration and individual development were prioritised over sustained relational and bespoke support. Figure 4 shows support mechanisms.

#### Relational support and responsive programme design

Practitioners emphasised support as relational, continuous and responsive, rather than as a one-off response to crises. Meeting young people “where they’re at” captured practical flexibility and sensitivity to emotional and social contexts (energy levels, confidence, trauma, competing demands, and cultural realities):

“[its asking] like what you bring into the course today?… What do you want us to be aware of?…What’s your learning style? What is your comfort level on this and the other?… [after that] I had a really good idea of…. what type of learners I’ve got. [and the] Sensitivity issues that they wanted me to be aware of” [Workforce/skills leadership].

Support was Support was understood as essential for leadership to be possible in community contexts, especially for underserved girls juggling access barriers (caregiving responsibilities, precarious work and cultural expectations):

“They need consistency… how can we link them up with a mentor? Someone who really understands the barriers they’re facing…” [National Women’s sport Development/programme officer].

Digital platforms like WhatsApp extended peer support and connection beyond programme sessions:

“We build communities of women… [using] WhatsApp community…[so] when they are having problems… they have that community around them.” [Workforce/skills lead].

These accounts align with CPYD’s emphasis on supportive environments as redistributive resources for young people facing intersecting inequalities.

#### Practitioner visions for more relational and representative leadership systems

Practitioner

Top-down recruitment and standardised delivery were challenged as restrictive and exclusionary. Practitioners emphasised peer-led, culturally credible outreach and flexible, co-designed delivery approaches. One faith/community leader, explained: “*Have consultation groups… and ask them to co-design the way forward… the delivery [approach]*,* the time*,* the length*.” Hybrid models (online and in-person) were reduced structural barriers such as childcare and transport: “*Blended learning… stops barriers of childcare and travel”* [Workforce/skills lead]. Young women were positioned not simply as programme recipients, but as co-creators of their own leadership journeys, with support conceptualised as relational infrastructure: “*building communities of women… not just a programme but [plugging] them into a community [is important]*” [Workforce/skills lead] was central. Participants rejected transactional models for embedded support networks extending beyond programme completion.

Structural representation was both a barrier and a lever for change, and the lack of female coaches and role models was widely noted: “*There just isn’t enough female coaches to train other women*” [Community/grassroots practitioner]. Yet, positive effects were described when female leadership was supported: “*Women supporting women*,* supporting girls… it’s a really lovely pass back that seems to happen*” [Workforce/skills lead]. Participants called for investment in representation, visibility, and sector-wide reform :

“Being able to see the next step up… shared experience of a leader who’s in journalism or national policy… So these women can see there are so many different avenues [to be a leader]” [Workforce/skills lead].

#### Navigating structural barriers through cultural and relational adaptation

Practitioners highlighted tensions between relational intentions and structural constraints. Intensive delivery formats, inflexible structures, and a lack of female facilitators limited access:

“We did the full 30 hours within a week… it was too much for everybody… especially having an SEN [special educational needs] person on that as well.” [Community/grassroots practitioner].

“We don’t have any female leaders… that can deliver this… [having a male coach] was a barrier. [because some cultures require female staff]” [Faith/community leader].

Limited funding meant gender-matched or bespoke provision was often not feasible:

“We would need far more resources than what we’ve got… targeted interventions would be a whole different project [to deliver].” [Senior leadership].

Yet sustained trust-building and cultural-attunement could mitigate some barriers, including gender-matching:

“It’s about building that trust and engaging with them positively right from the beginning… to know what’s going to work for them.” [Faith/community leader].

Yet, trust through relationality was described as sometimes mitigating the need for gender matching, particular when there were cultural norms at play such as for Muslim populations:

[Ive had a different experience] We went to a local mosque. We engaged with that mosque… [, we built up the trust… [then they said we can deliver] But these are going to be the requirements. This is the sort of cultural nuances. This is what …[we] need. And then we tailored the programmes to fit the girls and then from there… [we] looked at upskilling and how you can qualify them to be leaders and inspire other girls from that community. [Senior leadership]

These examples illustrate the labour of making leadership development culturally resonant, and the ways in which practitioners work within and around structural constraints.

#### Incentivising inclusion: financial support as enabler and tension

Financial supports (vouchers and payment) emerged as a powerful enablers with practical and symbolic weight.

It’s not about the money—it’s about what that money means. For some, it’s the bus fare. For others, it’s getting time off their zero-hours job.” [Senior leadership].

Practitioners emphasised that many girls would not attend without such support:

“We gave out vouchers… £10 for every session… that helped them stay on …Most of our girls wouldn’t be able to come… without us paying” [Community grassroots practitioner].

Financial support was also seen as potentially protective, offering alternatives to criminalised income-generation:

Money in these type of programmes really can make a difference in a young person’s life. You could really be taking them off the streets and away from getting into gang and into drugs and various different things just by offering them a set amount of money within. [Senior leadership]

However, tensions arose when payment was not attached to clear roles and responsibilities, and was critiqued for fostering unrealistic expectations:

“… [you need to] make sure that… they’re not just getting paid to just stand around and be an assistant coach like, give them responsibilities, let them lead sessions, like start trusting them… Because… I don’t know if it’s sort of teaching them the right thing because they’re going to go into the industry and have to run full sessions for this amount of money and [they will think]… wait, hang on”. [Senior leadership]

Overall, Theme 2 highlights support as relational, redistributive and culturally situated but continually negotiated within funding and organisational constraints.

### Theme 3: programme outcomes

**Fig. 5:**
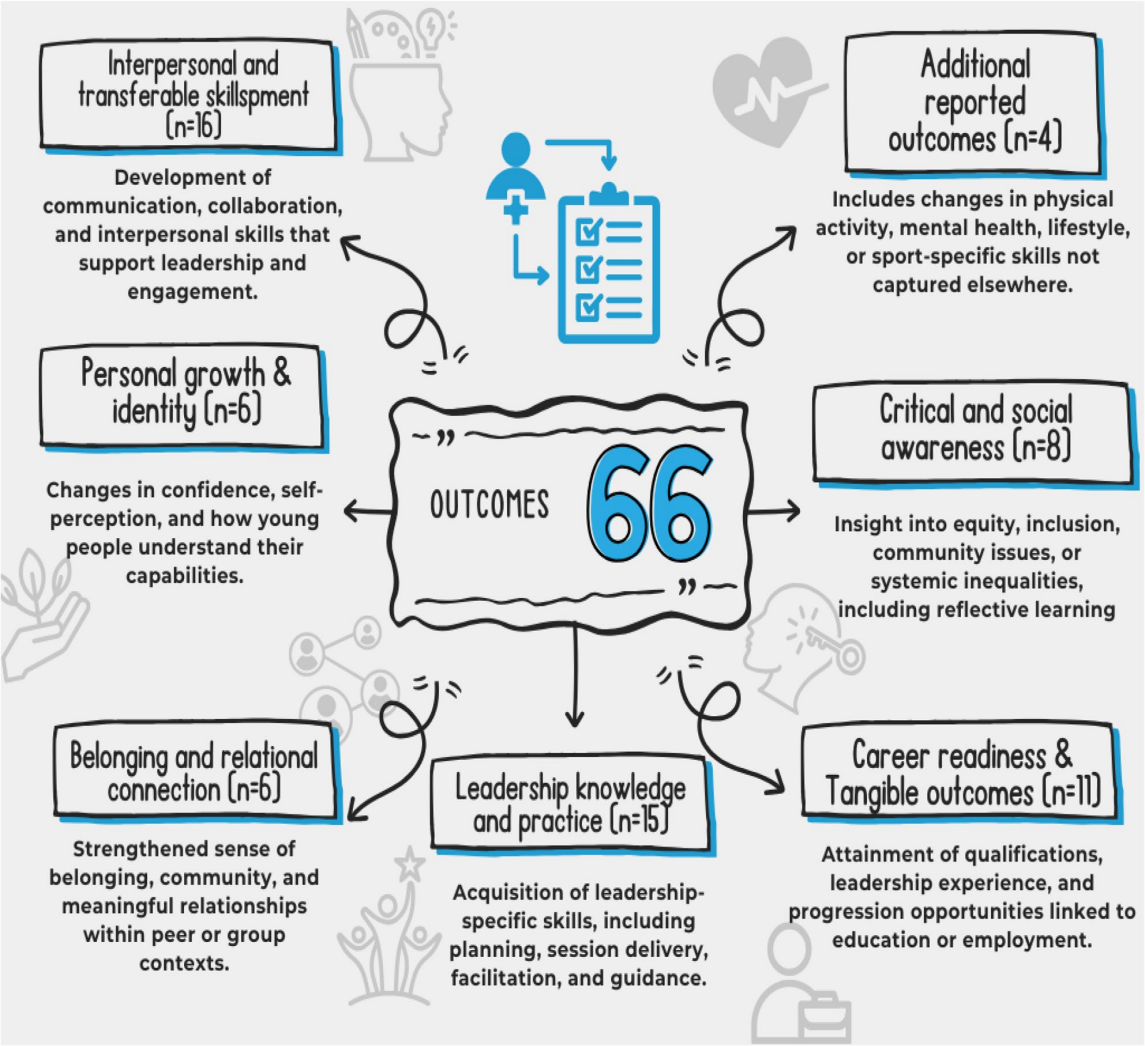
programme outcomes found during systematic mapping

Systematic mapping identified 66 outcome mechanisms across seven themes (Fig. 5). Most outcomes related to interpersonal and transferable skills, leadership knowledge and practical competence. Career readiness was also emphasised indicating a strong emphasis on employability. Fewer programmes highlighted personal growth, belonging, relational connection or physical and mental health, suggesting that wellbeing- and relationship-oriented outcomes are less prioritised than skill-based ones.

#### Personal and relational foundations of leadership

Practitioners framed personal growth as a foundational for leadership development, particularly for girls and young women rarely positioned as leaders. Programmes aimed to support confidence, agency, voice, and interpersonal development:

“Young girls’ voices being heard and empowerment of confidence… going from being the participant to being the active person standing in front of the participants”. [Workforce/skills lead]

“Seeing people being able to have their voices heard… that can provide lifelong skills… just trying to have the holistic improvement of the person…”[Senior leadership].

These outcomes were viewed as critical preconditions for sustained engagement in leadership and PA, forming the foundation of other leadership capacities. Relational development and belonging were equally vital, especially for those facing isolation. Practitioners emphasised building relational ecosystems around young women, rather than expecting them to “stand alone” as leaders, using peer structures and digital platforms to foster ongoing connection and “build communities of women to support [one another]” [Workforce/skills lead]. Taken together, these accounts position personal growth and relational belonging essential foundations of leadership for underserved girls and young women.

#### From leadership as a role to an identity

Practitioners described leadership as something embodied in practice and personally meaningful, not symbolic. They reflected on how their understanding had shifted:

“When we were speaking about defining what a leader is… I’ve come in from the leader being like the coach. delivering sessions… [but I think its more than that]… its about not having expectations at the start [but allowing young people to make their own definitions of leadership]” [Community grassroots practitioner].

Young people’s sense of self as leaders was noted as emerging through leadership enaction, recognition and responsibility, and though being entrusted with meaningful tasks, becoming visible within their communities and being recognised by peers and adults. Leadership was outlined as an ongoing evolution of confidence, competence, and presence. Yet participants cautioned that identity development, without opportunity to enact leadership in consequential spaces, risked leaving programmes hollow.

#### Redefining leadership: representation, decision making and social change

Participants described a shift influence-based and advocacy-oriented models of leadership, that challenge narrow, transactional interpretations (e.g., coach development). Leadership was increasingly framed as a vehicle for systemic change, structural representation, and community advocacy. As one practitioner explained:

*We’ve identified [women] through relationships we’ve built over the last few years… [and we’ve asked] firstly [do] they want to be a leader… [the leadership were developing] is for leadership positions that are more from a broad level… we talk about defining leadership… this is where it is… It’s more sort of real decision maker… we’ll hopefully be able to diversify the workforce a little bit better to get people that really feel and understand the challenges at leadership level.* [Senior leadership]

Practitioners highlighted examples of young women, particularly from ethnically minoritised communities, acting as visible agents of change:

*“We’ve got young girls that are co-delivering the sessions… in some settings*,* they’re wearing the hijab*,* they’re representing their faith… it breaks down that stereotype of what a leader in sport looks like.”* [Workforce/skills lead].

Theme 3 traces a conceptual movement from leadership as skill accumulation to leadership as identity, representation and social change, aligning with CPYD’s emphasis on critical consciousness and with feminist–intersectional concerns about who is seen and who speaks in PA spaces.

### Theme 4: Recommendations for future practice

**Fig. 6:**
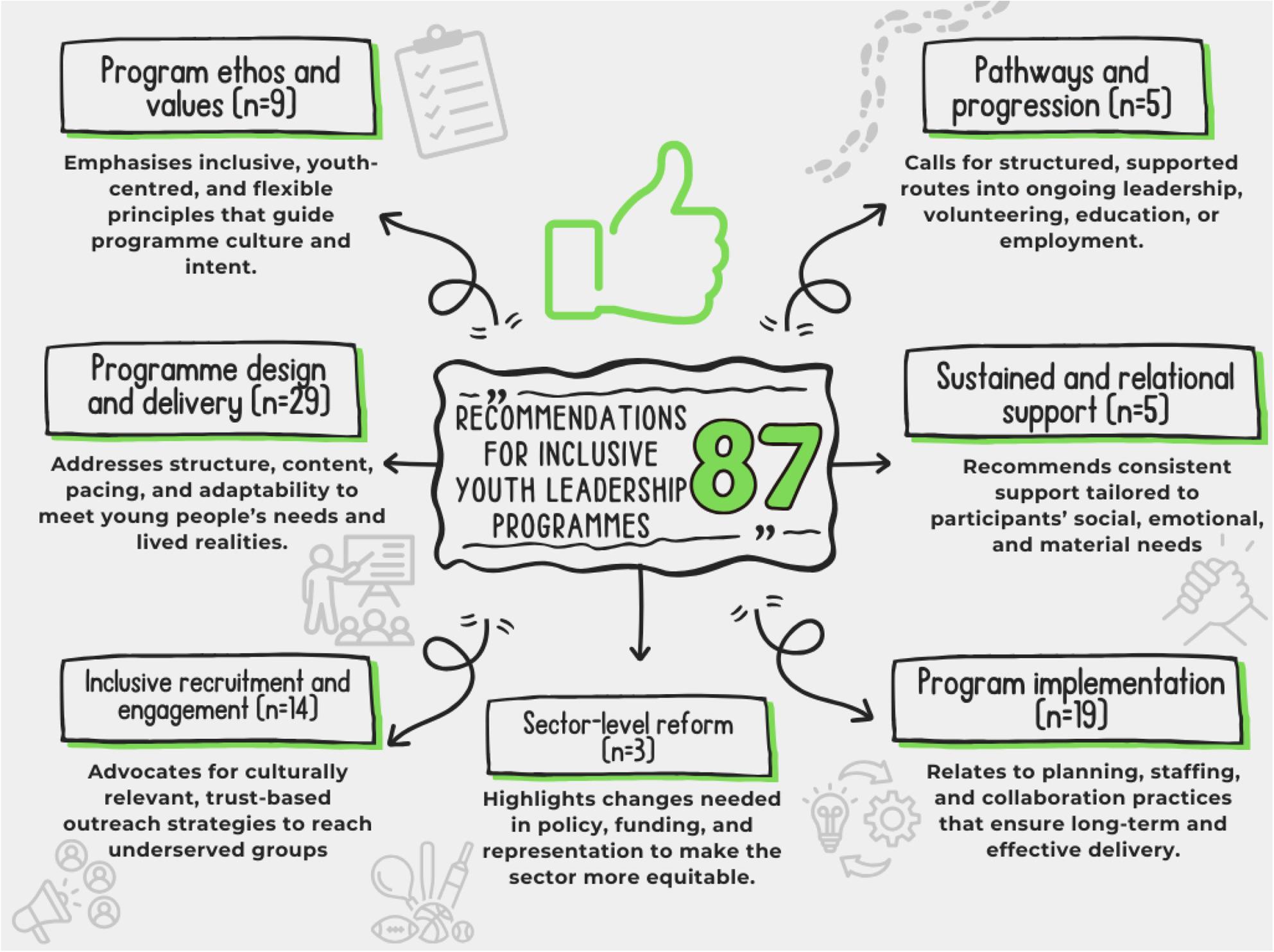
Recommendations for inclusive youth leadership programmes found during systematic mapping

Systematic mapping identified 87 practitioner-informed recommendations clustered into seven thematic areas (Fig. 6). Most focused on programme design and delivery, implementation and inclusive recruitment, with others addressing ethos, progression pathways, relational support and sector reform. Together, these mapped recommendations highlight a desire for accessible, responsive and equity-oriented leadership pathways.

#### Emphasising progression and sustainability

Practitioners stressed the need to decouple leadership from narrow coaching- and qualification-focused models, which alienate youth who did not identify with traditional leadership “*stereotypes*” (e.g., extroverted). Instead, participants advocated for reframing leadership as advocacy, ambassadorship, and influence rooted in cultural identity, lived experience and social change.

Practitioners stressed building real-world exit routes into ongoing leadership roles. Without these, programmes risk becoming symbolic rather than developmental: “[our programme was strong because] *There was tangible exit routes for [them]… [it’s] not just a one-dimensional project… there’s a pathway there for them to progress*” [Senior leadership]. The key recommendation was that youth leadership development programmes should be *‘more than just a course’ [Development/programme officer]* – but be nurturing, not merely transactional.

## Discussion

This study explored how PA leadership programmes engage underserved groups of girls and young women, and focused on identification, support, delivery, and progression. Drawing on systematic mapping and qualitative focus group data with practitioners, it contributes new knowledge to a relatively underexplored area: inclusive and equitable youth leadership in PA contexts. While leadership is increasingly positioned as a tool for fostering youth engagement and empowerment in sport and PA [7, 15], our findings show current practice often reproduces inequalities. To our knowledge, no previous study has systematically mapped PA leadership programmes targeting underserved girls and young women nationally while also incorporating in-depth practitioner perspectives. Existing work tends to focus on youth PA participation or on leadership generally, with limited attention to equity and little explicit use of CPYD and feminist–intersectional lenses.

### Programme visibility, investment and structural (in)coherence

Systematic mapping revealed 2017 and 2022 saw the most new programmes, reflecting a political and funding emphasis on youth leadership, gender equity and inclusion in PA. This included Sport England strategies (e.g. *This Girl Can*, 2016–21 [38]), Local Delivery Pilots [39], and the Youth Investment Fund [40]. However, few programmes were initiated after 2020, coinciding with the COVID-19 pandemic and subsequent funding volatility [39, 41, 42]. Despite rhetorical commitment to inclusion, programme documentation frequently referenced time-limited funding or pilot status, with few sustained or mainstreamed examples, indicating a fragility without sustained investment. From a CPYD perspective, this volatility undermined the stability needed to support ongoing leadership development for underserved girls and young women.

Variation in programme delivery models indicates a broader lack of standardisation across the youth PA leadership sector. Programme flexibility enabling local adaptation, but reflected a pragmatic landscape with limited coherence or shared principles. Mapped programmes varied markedly in duration, intensity, entry criteria and progression routes, and few articulated equity or intersectional design principles. We observed a double bind in that programmes attached to education, or partner organisations risked activating the Matthew Effect [43], where advantaged groups access further opportunities while underserved groups remain overlooked. The further bind being that those opportunities embedded in wider systems were vulnerable to inconsistent funding and implementation.

### Recruitment and identification

Mapping recorded diverse recruitment mechanisms, including partnerships, education-based outreach, and digital campaigns. However, focus groups revealed tension between methods and values. Despite efforts to increase reach, many methods disproportionately accessed already-resourced, confident and connected young people and failed to apply an intersectional lens. Digital outreach was limited by linguistics, imagery and digital poverty. More equitable recruitment was rooted in relational, culturally competent, and intersectional sensitive outreach, with trust being as key.

These findings imply that recruitment cannot be detached from wider programme design. To be equitable, leadership pathways must centre youth, community, and culturally relevant leadership. An intersectional lens shows how reliance on institutional networks, digital outreach and English-language materials disproportionately benefits those already advantaged while compounding barriers linked to poverty, disability and racialised marginalisation.

### Ethos, support and belonging

Support to sustain engagement and develop leadership skills was considered central to programme success, framed as both a recruitment and retention mechanism and a condition for making leadership possible in community contexts. Care-oriented support mechanisms, such as connection to wider support networks and peer WhatsApp groups, were widely acknowledged as impactful. Flexible delivery models were vital to allow underserved young people to engage in relevant ways. Critical Positive Youth Development [17] directs us to view these support practices as redistributive: mechanisms that reallocate time, attention and resources towards young people whose leadership opportunities are constrained by competing responsibilities.

Belonging emerged as a particularly important outcome and a condition for development. Respondents described how inclusive environments allowed girls to challenge gendered and cultural stereotypes, and view themselves as leaders. But belonging needed to be purposively designed, not assumed and embedded in programme ethos. Intersectional perspectives underscore that belonging must address how gender, ethnicity, religion, disability and class shape who feels ‘at home’ in leadership spaces. This points to the wider politicisation of support: programmes must sustain individual engagement but *and* reshape the PA system so underserved young women see themselves reflected in its leadership structures. Such transformation requires visibility, investment, and a commitment to changing who leads and how.

### Conceptualising youth physical activity leadership: from coaching to community influence

Practitioners cited common constructions of youth leadership within PA as narrow, adult-centric and oriented around credentialism. We observed leadership being used as a proxy for coaching, with systematic mapping defining leadership via coaching pathways and formal qualifications. While these offer legitimacy and progression for some, they risk excluding young people whose leadership is informal, relational, or culturally situated. In contrast, focus groups, highlighted an emerging drive towards advocacy-based and influence-focused leadership models challenging transactional definitions. Particularly for underserved groups of young women, leadership can be conceptualised via cultural representation, care, relational and systems-level influence.

These findings offer deeper understanding of those presented in previous research [7, 22] and reflect critical positive youth development literature, positioning leadership not merely as skill acquisition but as social leveraging and political voice [17]. Whilst there is desire to implement such approaches, the contrast between mapping and focus group data suggests they remain the exception rather than the rule, often relying on individual practitioners. Feminist and intersectional leadership perspectives help to interpret these shifts as moves towards recognising care, cultural representation and community advocacy as legitimate forms leadership.

### Progression and continuity

Pathways to further opportunities were often left to individual effort. Leadership programs must shift sustained engagement as a systemic priority. This echoes broader concerns that without intentional progression structures, leadership programmes risk becoming one-off experiences that do little to shift longer-term representation or access to decision-making roles [22]. Many respondents were critical of short-term models lacking progression routes into meaningful opportunities. However, progression needs to be diverse. Practitioners called for visible, culturally relevant progression routes, and for leadership to be understood as a continuous journey. Enhancing leadership development requires programme-level improvements and system-level changes that secure continuity and embed progression. Programmes should support underserved young people to enter the PA leadership workforce, and both navigate and challenge the system that supports their marginalisation.

### Intersectionality and structural change

Despite growing rhetoric around inclusion, most programmes did not embed intersectionality in their design [18]. Gender and ethnicity were considered, but complex interconnected factors like disability, socio-economic status or neurodivergence, were often underacknowledged. In our mapping, intersectional recruitment was rare, with programmes often targeting broader categories such as ‘girls’ or ‘Lower Socio-economic Group’ than specific, intersecting experiences. Subsequently, inclusion remained surface-level. Focus group participants advocated for designs beginning with lived experience and reflecting what leadership means for individual young people and local communities. This challenges the sector to value alternative leadership expressions and embed intersectionality as a foundational.

### Strengths and limitations

This study’s key strength is its integrated design, combining systematic mapping with practitioner focus groups. Mapping provided a structured overview while focus groups added depth, allowing us to interrogate equity implications rather than only catalogue approaches. However, several limitations should be noted. First, programme identification relied on organisational visibility and existing networks, meaning informal, small-scale or emerging initiatives may be missed and mapping likely over-represents well-established, well-resourced and digitally visible programmes. Much of the mapping data was drawn from publicly available or self-reported descriptions, which varied in depth and may not fully capture how programmes operate(d). Second, although we purposively sampled practitioners from diverse roles and organisations, the focus group sample included relatively few practitioners from racially minoritised backgrounds or with lived experience of disability, which may have shaped prioritised issues. Finally, the work is embedded in the UK policy and funding context, and while many insights are conceptually transferable, specific structures and mechanisms may differ elsewhere.

## Conclusion

While systematic mapping provided breadth of descriptive insight, focus group data added depth, together illustrating how values and delivery interact. Mapping identified recruitment strategies, but focus groups revealed these often reproduce exclusion without cultural awareness and intersectionality. Mapping showed support mechanisms, but focus groups highlighted support’s dependency on individual rather than structural commitment.

Together the datasets reveal both potential and pitfalls of current provision. They outline sector wide intent for inclusion yet highlight practice constraints. Leadership programmes can support inclusion and representation in PA, but without addressing structural, conceptual, and cultural limitations, they risk reinforcing inequalities.

Regarding Aim 1 (mapping current provision and practices), our analysis shows leadership programmes for underserved girls and young women are well-intentioned but have fragile structures: (1) recruitment is mediated by institutional gatekeepers and digital access; (2) support is relationally rich but structurally under-resourced; (3) outcomes focus on skills and employability; and (4) progression routes are patchy and largely rely on individual effort. In relation to Aim 2 (practitioner recommendations), practitioners called for: (i) relational and culturally grounded recruitment; (ii) care-oriented, redistributive support; (iii) broader leadership conceptualisations valuing representation and advocacy, (iv) resourced progression pathways with sector-wide principles and longer-term investment.

Our study supports rejecting one-size-fits-all approaches. Instead, programmes should embrace youth-led, context-sensitive practices treating leadership as a process of becoming rather than an achievement checklist. We outline the following recommendations.

### Recommendations for research and practice

- Define leadership beyond credentialed coaching to include advocacy, cultural influence, and relational care.
- Embed intersectionality in programme design, recognising how multiple identities shape leadership potential and experience.
- Resource progression pathways to ensure sustainability and meaningful development.
- Invest in structural support, not just individual delivery, to embed care, access, and belonging.
- Use youth-defined frameworks to co-design leadership programmes reflective of lived realities.
- Develop sector-wide principles that balance flexibility with coherence.

## Data Availability

Datasets are not publicly available due to ethical restrictions, but are available from the corresponding author on reasonable request.

## Abbreviations

PA: Physical activity
CPYD: Critical Positive Youth Development
